# Head, Hands, Knees and Ankles, Knees and Ankles: Injury Profiles of Women and Girls Playing Community Australian Football

**DOI:** 10.1177/19417381241303512

**Published:** 2025-02-04

**Authors:** Sallie M. Cowan, Brooke E. Patterson, Matthew G. King, Mick A. Girdwood, Andrea B. Mosler, Alex Donaldson, Adam G. Culvenor, Andrea M. Bruder, Melissa J. Haberfield, Michael Makdissi, Christian J. Barton, Eliza Roughead, Sarah Lampard, Karina Chilman, Christian Bonello, Elizabeth Birch, Joshua Frost, Deirdre E. McGhee, Kay M. Crossley

**Affiliations:** La Trobe Sport and Exercise Medicine Research Centre, La Trobe University, Victoria, Australia, and Australian IOC Research Centre, La Trobe University, Melbourne, Victoria, Australia; La Trobe Sport and Exercise Medicine Research Centre, La Trobe University, Victoria, Australia, and Australian IOC Research Centre, La Trobe University, Melbourne, Victoria, Australia; La Trobe Sport and Exercise Medicine Research Centre, La Trobe University, Victoria, Australia, Australian IOC Research Centre, La Trobe University, Melbourne, Victoria, Australia, and Discipline of Physiotherapy, School of Allied Health, Human Services and Sport, La Trobe University, Victoria, Australia; La Trobe Sport and Exercise Medicine Research Centre, La Trobe University, Victoria, Australia, and Australian IOC Research Centre, La Trobe University, Melbourne, Victoria, Australia; La Trobe Sport and Exercise Medicine Research Centre, La Trobe University, Victoria, Australia, and Australian IOC Research Centre, La Trobe University, Melbourne, Victoria, Australia; Australian IOC Research Centre, La Trobe University, Melbourne, Victoria, Australia, and Centre for Sport and Social Impact, La Trobe Business School, La Trobe University, Melbourne, Victoria, Australia; La Trobe Sport and Exercise Medicine Research Centre, La Trobe University, Victoria, Australia, and Australian IOC Research Centre, La Trobe University, Melbourne, Victoria, Australia; La Trobe Sport and Exercise Medicine Research Centre, La Trobe University, Victoria, Australia, Australian IOC Research Centre, La Trobe University, Melbourne, Victoria, Australia, and Discipline of Physiotherapy, School of Allied Health, Human Services and Sport, La Trobe University, Victoria, Australia; La Trobe Sport and Exercise Medicine Research Centre, La Trobe University, Victoria, Australia, and Australian IOC Research Centre, La Trobe University, Melbourne, Victoria, Australia; La Trobe Sport and Exercise Medicine Research Centre, La Trobe University, Victoria, Australia, and Australian IOC Research Centre, La Trobe University, Melbourne, Victoria, Australia; La Trobe Sport and Exercise Medicine Research Centre, La Trobe University, Victoria, Australia, Australian IOC Research Centre, La Trobe University, Melbourne, Victoria, Australia, and Discipline of Physiotherapy, School of Allied Health, Human Services and Sport, La Trobe University, Victoria, Australia; La Trobe Sport and Exercise Medicine Research Centre, La Trobe University, Victoria, Australia, and Australian IOC Research Centre, La Trobe University, Melbourne, Victoria, Australia; La Trobe Sport and Exercise Medicine Research Centre, La Trobe University, Victoria, Australia, and Australian IOC Research Centre, La Trobe University, Melbourne, Victoria, Australia; La Trobe Sport and Exercise Medicine Research Centre, La Trobe University, Victoria, Australia, and Australian IOC Research Centre, La Trobe University, Melbourne, Victoria, Australia; La Trobe Sport and Exercise Medicine Research Centre, La Trobe University, Victoria, Australia, and Australian IOC Research Centre, La Trobe University, Melbourne, Victoria, Australia; La Trobe Sport and Exercise Medicine Research Centre, La Trobe University, Victoria, Australia, and Australian IOC Research Centre, La Trobe University, Melbourne, Victoria, Australia; La Trobe Sport and Exercise Medicine Research Centre, La Trobe University, Victoria, Australia, and Australian IOC Research Centre, La Trobe University, Melbourne, Victoria, Australia; Breast Research Australia, University of Wollongong, Victoria, Australia; La Trobe Sport and Exercise Medicine Research Centre, La Trobe University, Victoria, Australia, and Australian IOC Research Centre, La Trobe University, Melbourne, Victoria, Australia

**Keywords:** ACL, concussion, female athlete, head injury, knee injury, sport

## Abstract

**Background:**

Women’s participation in all football codes (including Australian Football [AF]) is increasing rapidly. To guide injury prevention strategies, the authors aimed to describe the current and lifetime prevalence of significant musculoskeletal injuries and concussions for women and girls playing community AF.

**Hypothesis:**

Women will have high rates of injury associated with playing AF.

**Study Design:**

Cross-sectional survey.

**Level of Evidence:**

Level 3.

**Methods:**

Participants were Victorian community AF players from 165 participating teams (<16 years, <18 years, senior women’s). Demographics, injury prevalence, and health outcomes are reported descriptively. To explore relationships between sociodemographic factors and anterior cruciate ligament (ACL) injury history, the authors fitted univariate logistic regression models. Independent variables were age, body mass index, number of career AF games, sport experience, location (metropolitan/regional), and socio-economic index.

**Results:**

A total of 2435 players (95% of players enrolled in the trial), aged 24 ± 7 years completed the survey. One-quarter (n = 619, 25%) reported a current injury, and half (n = 1238, 51%) reported a previous significant injury. The most common injury sites were knee (n = 160 26% current, n = 403 33% previous), ankle (n = 130 21% current, n = 427 35% previous), and hand/fingers (n = 100 16% current, n = 317 26% previous). Self-reported previous ACL injury (n = 139, 6%) and concussion (n = 1335, 55%) were also prevalent. Increasing age (odds ratio [OR], 1.07; 95% CI, 1.05-1.09) and more career games (OR, 2.22; 95% CI, 1.24-3.97) were associated with ACL injury history.

**Conclusion:**

Women and girls playing community AF reported high rates of significant injury. Injury prevention programs should target the most prevalent injury sites: head (concussion), ankle, knee, and hand/fingers.

**Clinical Relevance:**

These findings highlight high injury rates for women playing AF and will be invaluable in shaping injury prevention strategies.

Women’s and girl’s participation in Australian Football (AF) has increased dramatically since the inception of the Australian Football League Women’s (AFLW) elite competition in 2017.^11^ Between 2016 and 2018, women’s community club teams grew by 137% (960 teams to 2281 nationwide),^6,7^ women participants grew by 87% between 2015 and 2022, and women made up 31% of all participants in 2022.^5,8^ The AFLW competition is now Australia’s largest employer of women athletes.^8^ This rapid evolution is exciting and significant for Australian women’s sport but has been accompanied by media attention and concern around the risk of significant knee injuries and concussion for women playing AF.^1,12,35^ Despite much speculation and assumption that these rates are similar for women playing community AF, we know little about their injury profile.

Knee and ankle ligament sprains have been reported as the most prevalent injuries in community women’s AF.^22,23^ Before the inception of AFLW, these data were from emergency department presentations and insurance data.^22,23^ The rise in AF participation in all age groups and competitive levels warrants further evaluation of significant injury in community women’s and girls’ AF. A more recent prospective cohort study confirmed that lower extremity injuries were most common^19^ and noted higher incidence rates for women and girls playing elite compared with community AF; however, the small sample (n = 257) of community players limits the generalizability of these findings and highlights the need for further research.

Women who play team-based collision sports have up to double the risk of an anterior cruciate ligament (ACL) injury or concussion^29,33,42^ and experience worse recovery (eg, worse symptoms, reduced return to sport) compared with men.^13,42^ Injury risk may be exaggerated in AF due to the full body contact, high intensity change of direction, and multidirectional game play unique to this code.^14^ For women new to AF, lack of exposure to training, and later or delayed technique development, might compound these risks. Other contextual (eg, level of competition) and sociodemographic (eg, socio-economic status, other sports participation) factors may also influence injury risk and recovery but have been rarely investigated. Understanding the injury prevalence and the associated contextual and sociodemographic characteristics will guide what and who to target with future injury prevention strategies. We aimed to describe the current and lifetime prevalence of significant musculoskeletal injuries and concussions in women and girls playing community AF.

## Methods

### Study Design

This survey was conducted in accordance with the Checklist for Reporting Results of Internet E-Surveys (CHERRIES) aimed at improving the quality of surveys conducted on the Internet (Appendix 1, available in the online version of this article).^18^ This was a cross-sectional survey (December 2020-May 2021) administered before the interventions for a stepped-wedge, cluster randomized trial measuring the effectiveness of an injury prevention program for women and girls playing community AF.^31^ Ethical approval was granted by La Trobe Human Research Ethics Committee (HEC 20488).

### Recruitment

Recruitment for our study began at a team level; coaches of women’s community AF teams consented to participate in the clinical trial and invited the players in their team to complete the survey. To be included, players had to be at least 13 years of age and be able to understand written English. All players provided electronic written informed consent before participating. All teams competing in under 16 (U16), U18, or senior women’s leagues in metropolitan (n = 9 leagues) and regional (n = 7 leagues) Victoria in Australia were invited to participate. Coaches were informed about the study via email from their club, or league, or through visits to team training from the research team. Leading officials, players, coaches, and partner organizations supported recruitment via social and mainstream media. Teams were included if (1) they competed in an organized senior (excluding masters) or junior (U16, U17, U18, Youth) league; (2) they trained at least once per week; and (3) the coach consented for the team to participate (including willingness to be supported to implement an injury prevention program (Prep-to-Play) in 2021 and/or 2022).^31^ A total of 165 teams agreed to participate before the 2021 season. A total of 5182 players were registered to play in 2021 from the 165 teams.

### Outcomes

The player survey, completed online via the secure REDCAP platform,^25^ included questions about current and lifetime prevalence of injuries, medical and women’s health factors, sociodemographic, and contextual factors (Appendix 2 - Player Survey, available online).

#### Current and Lifetime Prevalence of Injuries

Current injuries (point prevalence at the time of completing the survey) and previous significant injuries (lifetime prevalence) were calculated for the following body sites: head, neck, shoulder, elbow, hand/fingers, back, hip/groin, quadricep, hamstring, knee, ankle, calf, and foot. To estimate current injury prevalence, players were asked: “Do you currently (during the last week) have an injury, and/or pain that has limited your full participation in training or games?” To estimate lifetime prevalence of significant injuries players were asked: “Have you ever had an injury that resulted in you missing football (or other sport) training/games for more than a month (4 weeks)?”^24^ As 2 of the most burdensome injuries in sport, additional information about previous ACL injuries and self-reported suspected concussions was collected.^37^ Players were asked about lifetime history of ACL injury, whether they had undergone reconstruction surgery, and family history of ACL injuries. A standardized practical definition of concussion was provided to participants to assist in the reporting of suspected concussions. The definition included head impact that caused the player to be “knocked out cold or unconscious” (self-reported concussion with loss of consciousness [LOC]), as well as collisions and/or head impact that resulted in the player being briefly dazed or stunned or experience dizziness, confusion, balance problems, blurred vision, slowed reactions, nausea, difficulty concentrating, or headaches (self-reported concussion without LOC). We asked the number of concussions that the player experienced (1) playing AF, (2) playing other sport, or (3) in nonsport related settings.

#### General Health Factors

Players were asked to indicate whether they had been diagnosed with any of the following medical conditions: diabetes, asthma, heart conditions, high blood pressure, lung conditions other than asthma, polycystic ovarian syndrome, endometriosis, or cancer, and to provide details of any other medical conditions. Players could opt out of this question if desired.

#### Sociodemographic and Contextual Factors

Players were asked to provide sociodemographic factors, including age, residential postcode, occupation, employment status, level of education, self-reported physical characteristics (height, body mass), and current or past participation in other sports and level of competition (international, national, state, or local/school). They were also asked to report their highest level of AF experience (national, state, community/local/school) and how many games of AF they had played in their career (0-10, 11-50, 51-100, 101-150, >150 games).

### Data Analysis

All injury outcomes, medical and women’s health factors, and sociodemographic characteristics were collated and summarized as frequencies and percentages or means and standard deviations as appropriate. For frequencies, to provide an estimate of uncertainty, we calculated CIs using the Clopper-Pearson method. We also presented the sociodemographic factors of interest by the subgroups of interest: (1) previous ACL injury; (2) previous significant knee injury (missing ≥4 weeks of training/games in any sport); (3) self-reported suspected concussion (in any sport or nonsport related); and (4) no injury (ie, players who did not report a history of ACL, significant knee injury or concussion).

To summarize geographic data, we used players’ current postcodes to classify them as living in metropolitan or regional areas. To classify socio-economic status based on location, we used Australian Bureau of Statistics (ABS) Socio-economic Indexes for Area (SEIFA) data from 2021.^4^ These data are freely available, with indicators from 5-yearly national census data used to form summary measures of socio-economic status. For our analysis, we used the index of relative socio-economic advantage and disadvantage (IRSAD), using postcode information for each participant. We used IRSAD deciles as recommended by the ABS - an ordinal rank from 1 (least disadvantaged) to 10 (most advantaged).

We fitted a series of univariable logistic regression models to explore relationships between current sociodemographic factors and the dependent variables of previous ACL injury, significant knee injury, and self-reported suspected concussion history. The independent variables used were age, body mass index (BMI), highest level of AF (ordered factor), number of games played (ordered factor), previous participation in other competitive sports, previous participation in other football codes, location (metropolitan, regional), and socio-economic index (IRSAD decile). All analysis was conducted in R (R Foundation for Statistical Computing).^39^

## Results

### Participants

**Figure 1.**
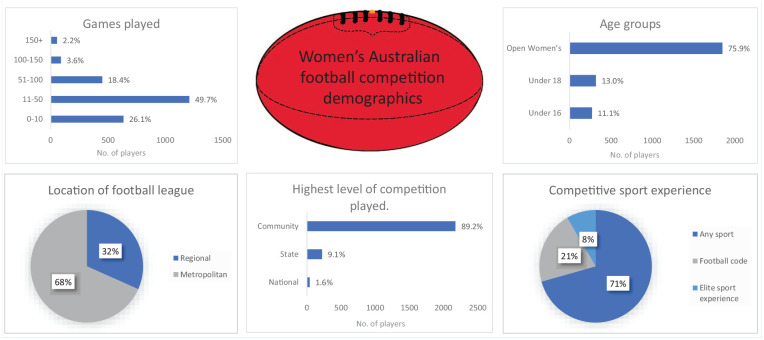
Women’s AF competition demographics. AF, Australian football.

A total of 2574 community women AF players consented to participate in the clinical trial,^31^ of which 2435 completed the survey for the current study, with 571 players (23.5%) <18 years of age. Demographic characteristics are listed in Table 1 and Figure 1. One-quarter of the players (26%) had played <10 games of AF and half (50%) had played between 11 and 50 games.

**Table 1.** Demographic characteristics of all participants

Variable	n = 2435
Age, y	24 (7)
Height, m	1.67 (0.07)
Body mass, kg	67.5 (13.2)
BMI, kg/m^2^	24.3 (4.4)
Aboriginal and/or Torres Strait Islander, n (%)	44 (1.8)
Gender identity, n (%)	
Woman/girl	2418 (99.1)
Nonbinary	10 (0.4)
Other	1 (0.04)
Not disclosed	3 (0.1)
Not reported	3 (0.1)
Employment status, n (%)	
Full time	1023 (42.0)
Part time	760 (31.2)
Not working	146 (6.0)
Home duties	52 (2.1)
Student full time	737 (30.3)
Student part time	123 (5.1)
Other	65 (2.7)

All values are mean (SD) unless otherwise specified. BMI, body mass index (based on self-reported height and weight).

### Current and Previous Injury Prevalence

**Figure 2.**
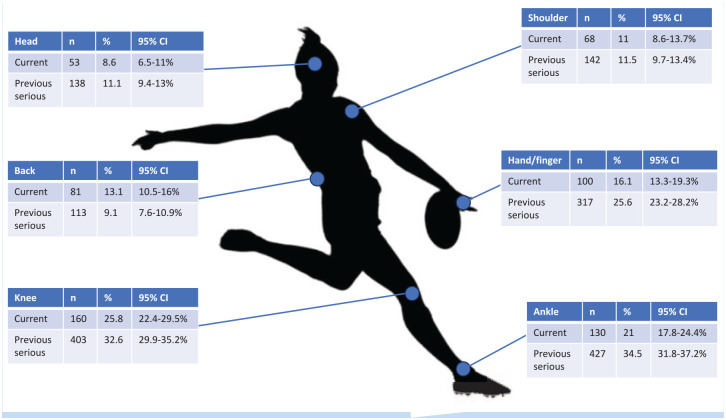
Most common self-reported current and previous serious injury prevalence by body region. See Table 1 in Appendix 3 for all regions prevalence (available online).

A total of 619 players (25.4%) reported a current injury impacting their participation in training and games, whereas 1238 (50.8%) reported a history of significant injury (Figure 2, and see Appendix 3 Table A1 for full list of injury prevalence, available online). The most common injury sites were the knee (25.8% of current injuries, 32.6% of previous significant injuries), followed by the ankle (21.0% of current injuries, 34.5% of previous significant injuries) and hand/fingers (16.1% of current injuries, 25.6% of previous significant injuries). Over half (54.9%) reported a suspected concussion without LOC, with almost one-third reporting a suspected concussion with LOC while playing AF (Table 2).

**Table 2.** Self-reported suspected concussion history

Self-Reported Suspected Concussion With LOC^a^	n	%	95% CI
AF	844	34.7	32.8-36.6
Other sport	725	29.8	28.0-31.7
Nonsport	776	31.9	30.0-33.8
Self-reported suspected concussion without LOC			
AF	1335	54.9	52.9-56.9
Other sport	920	37.8	35.9-39.8
Nonsport	877	36	34.1-38.0

a Participant response, n = 2433. AF, Australian Football; LOC, loss of consciousness.

#### Previous ACL Injuries

A total of 139 players (5.7%) reported a previous ACL injury, of which 113 had an ACL reconstruction (81% of ACL injuries). Increasing age (odds ratio [OR], 1.07; 95% CI, 1.05-1.09; Z = 6.11) was associated with previous ACL injury. Career games played showed a linear (OR, 2.16; 95% CI, 1.21-3.87; Z = 2.61) and quadratic (exponential) (OR, 1.85; 95% CI, 1.07-3.20; Z = 2.19) relationship to previous ACL injury. There was no evidence of a relationship for any other factor investigated (see Appendix 3 Tables A2-A4 for full model details for all outcomes, available online).

#### Previous Significant Knee Injuries

A total of 403 players (16.6%) reported a previous significant knee injury. Similar to ACL injuries, increasing age (OR, 1.03; 95% CI, 1.02-1.05; Z = 3.96), higher BMI (OR, 1.03; 95% CI, 1.01-1.06; Z = 2.733), and history of playing another football code (OR, 1.47; 95% CI, 1.16-1.85; Z = 3.24) were associated with a history of significant knee injury. Lower level of competition was associated with less previous knee injury (OR, 0.53; 95% CI, 0.33-0.85; Z = -2.62).

#### Previous Self-Reported Concussion History With LOC

A total of 1028 players (42.3%) reported a previous concussion with LOC (in any sport or nonsport related). Greater career games (OR, 1.74; 95% CI, 1.12-2.54; Z = 2.87), history of playing another competitive sport (OR, 1.64; 95% CI, 1.27-2.11; Z = 3.78), and history of playing another football code (OR, 1.29; 95% CI, 1.07-1.55; Z = 2.64) were associated with a previous self-reported concussion with LOC. Similar to significant knee injuries, we found playing at a lower level of competition was associated with reduced odds of self-reported concussion with LOC history (OR, 0.61; 95% CI, 0.39-0.95; Z = -2.18).

#### Associations With Sociodemographic Factors

We found no evidence of a relationship between postcode, socio-economic decile, and history of ACL, significant knee injury, or self-reported suspected concussion (Table 3). Living regionally was associated with higher odds of reporting a previous self-reported concussion with LOC (OR, 1.24; 95% CI, 1.05-1.47; Z = 2.47) compared with those living in metropolitan areas. The mean [SD] socio-economic decile was similar for all injury groups investigated (ACL injury, 7.18 [2.56]; significant knee injury, 7.27 [2.54]; concussion history, 7.40 [2.45]; no significant injury group, 7.41 [2.46]), and was not related to previous ACL, significant knee, or self-reported concussion history.

**Table 3.** Sociodemographic factors, split by injury history

	ACL Injury History (n = 139)			Significant Knee Injury History (n = 421)			Concussion History (n = 1020)			No Significant Knee/Concussion History (n = 1184)		
	N	%	95% CI	N	%	95% CI	N	%	95% CI	N	%	95% CI
Age group												
U16	4	2.9	0.8-7.2	27	6.4	4.3-9.2	92	9.0	7.0-10.9	159	13.4	11.5-15.5
U18	6	4.3	1.6-9.2	45	10.7	7.9-14.0	143	14.0	11.9-16.3	155	13.1	11.2-15.1
Senior	129	92.8	87.2-96.5	349	82.9	79.0-86.4	785	77.0	74.3-79.5	870	73.5	70.9-76
Overweight, >25 kg/m^2^	51	36.7	28.7-45.3	143	34.0	29.5-38.7	296	29.0	26.2-31.9-	320	2.70	24.5-29.7
Location												
Regional	92	66.2	57.7-74.0	289	68.6	64.0-73.1	668	65.5	62.5-68.4	833	70.4	67.7-72.9
Metropolitan	47	33.8	26.0-42.3	132	31.4	26.9-36.0	352	34.5	31.6-37.5	351	29.6	27.1-32.3
AF experience, games												
0-10	35	25.2	18.2-33.2	94	22.30	18.4-26.6	226	22.0	19.6-24.8	354	29.9	27.3-32.6
11-50	70	50.4	41.8-58.9	211	50.10	45.2-55.0	501	49.1	46.0-52.2	591	49.9	47.0-52.8
51-100	19	13.7	8.4-20.5	80	19.0	15.4-23.1	223	21.9	19.4-24.5	184	15.5	13.5-17.7
100-150	7	5.0	2.0-10.1	18	4.30	2.6-6.7	39	3.8	2.7-5.2	38	3.2	2.3-4.4
>151	8	5.8	2.5-11	18	4.30	2.6-6.7	30	2.9	2.0-4.2	17	1.4	0.8-2.3
Highest level of AF played												
National	4	2.9	0.8-7.2	13	3.1	1.7-5.2	23	2.3	1.4-3.4	13	1.1	0.6-1.9
State	23	16.5	10.8-23.8	54	12.8	9.8-16.4	125	12.3	10.3-14.4	69	5.8	4.6-7.3
Community	112	80.6	73.0-86.8	354	84.1	80.2-87.4	871	85.4	83.1-87.5	1102	93.1	91.5-94.5
Other sport playing experience												
Playing other sport	124	89.2	82.8-93.8	380	90.3	87-92.9	923	90.5	88.5-92.2	1006	85.0	82.8-87
Other football code^a^	43	30.9	23.4-39.3	138	32.8	28.3-37.5	303	29.7	26.9-32.6	262	22.1	19.8-24.6
Elite sport experience	21	15.1	9.6-22.2	55	13.1	10-16.7	125	12.3	10.3-14.4	106	9.0	7.4-10.7
Significant injury history												
ACL injury	-	-	-	139	33.0	28.5-37.7	62	6.1	4.7-7.7	-	-	-
Significant knee injury	-	-	-	-	-	-	192	18.8	16.5-21.4	-	-	-
Self-reported concussion with LOC	62	44.6	36.2-53.3	192	45.6	40.8-50.5	-	-	-	-	-	-

a Other football codes: rugby, soccer, and Gaelic or American football. ACL, anterior cruciate ligament; AF, Australian Football; LOC, loss of consciousness.

### General Health Factors

Self-reported general health factors are detailed in Table 4 with the asthma being the most prevalent self-reported comorbidity.

**Table 4.** General health factors

Self-Reported Comorbidities (n = 2421 Responders)	n	%	95% CI
Diabetes	11	0.5	0.2-0.8
Asthma	480	19.9	18.3-21.5
Heart condition	19	0.8	0.5-1.2
High blood pressure	16	0.7	0.4-1.1
Lung condition	8	0.3	0.1-0.7
Polycystic ovarian syndrome	91	3.8	3.0-4.6
Endometriosis	70	2.9	2.3-3.6
Cancer	1	0.1	0.0-0.2
Other illness	1801	74.5	72.7-76.2

## Discussion

This study profiled the largest cohort yet of women and girls playing community AF. The data demonstrate a substantial injury burden for women playing community AF, with one-quarter reporting a current injury, and over half reporting a previous significant injury. The most common current and previous musculoskeletal injury sites were knee, ankle, and hand/fingers. Previous self-reported suspected concussion was also prevalent (54.9%). These data are an important first step to understand injury profiles for women and girls playing community AF and will inform the design of future appropriately targeted injury prevention strategies.

### Overall Injury Prevalence

Injuries to the lower extremity were the most prevalent injuries reported by women and girls playing community AF in this study, consistent with previous studies in women’s and men’s AF.^19,21-23,38^ Specifically, the knee was the most prevalent current injury site and the second most prevalent previous significant injury site (32.6%), behind ankle (34.5%). This is in line with media attention and concern regarding the high rate of ACL injuries in elite women’s AF; however, the self-reported lifetime prevalence of ACL injury was relatively low in the present study (5.7%), considering other reports of ACL injuries per season ranges from 1% to 5% in other sports.^34^ This low lifetime prevalence may reflect the cross-sectional nature of our study and the fact that around 50% of players who suffer an ACL injury do not return to sport^3^ or have reduced exposure to AF and potential ACL injury due to the relative newness of AF as a sport for women. Hand/finger injuries were the upper extremity injury site with the highest prevalence (16.1% of current and 25.6% of previous significant injuries), consistent with previous studies of women’s community AF^19^ but different from men’s community and elite AF, where the shoulder is the upper extremity injury site with the highest prevalence.^21,38^ This may reflect a lack of AF-specific experience and may highlight the importance of improving ball handling (eg, marking, picking up and catching the ball, tackling). Interestingly, hand/finger injuries have lower prevalence than in other women’s football codes^27,32^ but are similar to netball (15%) and basketball (10%),^2,7^ which may reflect the unique nature and context of women’s community AF. These findings highlight the need for sport- and gender-specific injury prevention strategies.

### Concussion

The data indicate that concussion may present a significant burden for women playing community AF, with over half of our sample (54.9%; n = 1337) of players reporting a history of suspected concussion without LOC and over one-third (34.7%; n = 845) reporting a history of suspected concussion with LOC. This concurs with a previous small self-reported survey (33%) in community women’s AF^19,22,23^ and is higher than concussion levels reported in men’s community AF.^21,28^ Higher levels of concussion in women have also been reported in other ball sports.^16,28^ The high levels of suspected concussion reported in our study may also indicate increased community awareness about concussion and its potential serious consequences. In the past, many concussions were not reported,^26^ which may have been associated with poor concussion-related knowledge.^20,30,41^ The results of this survey suggest that concussion in women and girls playing community AF warrants further investigation to better understand the number, mechanism, and potential prevention strategies. It is important that coaches, trainers, and players recognize the significance of a concussion diagnosis for women and girls, follow the AFL concussion guidelines,^9^ and understand that it may take longer for women to fully recover from a concussion and return to play than men.^23,42^

### Injury-Associated Factors

Increasing age, more games played, and higher BMI were associated with a history of a significant knee injury. Previous self-reported suspected concussions were associated with greater numbers of career games, a history of playing another competitive sport, and living in a regional area. In our study, playing at lower levels of competition (in the community) was associated with history of self-reported suspected concussion without LOC and less significant knee injury history, which concurs with research indicating greater injury rates for women playing AF in matches and training at the elite level compared with the community level.^19^ It is possible that the increased injury rates relate to greater exposure to training sessions and games compounded with higher intensity of play (eg, greater running speeds, collisions) and therefore heightened injury risk with higher levels of competition.^10,17^

History of ACL, significant knee injury, or self-reported suspected concussion injury were not associated with the socio-economic decile of residence nor were there any differences between metropolitan and regional locations for any injuries investigated. This contrasts with other nonsport injury studies that report increased injury risk/poor health outcomes for those in the lower socio-economic decile of residence.^36,40^ Obesity as an important risk factor for knee injury. Although we found a higher BMI was associated with a history of significant knee injury, BMI was not associated with socio-economic decile of residence, and higher BMI may be a consequence rather than a cause of the significant knee injury.^43^ Alternatively, it is possible that, as we used current location for socio-economic data, it did not reflect the location where the player was born and raised.

### Clinical Implications

Women playing AF experience significant injury burden, with lower extremity injuries (knee and ankle), finger/hand, and concussion being most prevalent. The results highlight a different injury profile for women playing AF compared with men, emphasizing the need to design injury prevention programs specifically targeting women playing AF. The prevalence of finger and hand injuries is unique to women, warranting consideration in the design of programs. For women new to AF, these body regions may be at higher risk due to a lack of training and technique development for the unique demands of AF compared with other cutting/pivoting sports, for example, tackling/contact/falling skills (fall and concussion risk) or marking, and picking up the oval-shaped ball (for hand/finger injuries). It is also apparent that all ages (juniors and seniors) and both regional and metropolitan teams are in need of injury prevention strategies, with higher levels of competition potentially posing a greater risk. Another practical implication to consider when implementing injury prevention programs is the finding in our data that one-quarter of the team will have a current injury, potentially making adherence to injury prevention programs challenging.

### Strengths and Limitations

The most obvious limitation of this study is the self-reporting of injuries, with no medical diagnosis, as self-reported data have inherent limitations such as recall bias. Self-reported recall of concussions under reports actual concussions^15^; however, women may be less likely to under-report than men.^16^ In addition, as we did not collect exposure data (eg, time spent playing and training), it was not possible to calculate injury incidence, making direct comparison with some previous research difficult. Despite these limitations, the present study is, to our knowledge, the largest study of injury prevalence in women and girls playing community AF, with a wide range of ages, football and other sport experience, locations (regional and metropolitan), and competition level. Our study also provides a holistic assessment of medical and musculoskeletal conditions, improving our understanding of other factors relating to their health, context, and sociodemographic that may need to be considered when designing and delivering interventions (eg, 0.4% identified as nonbinary).

## Conclusion

This study explored current injury prevalence and injury history in a large (n > 2500) representative cohort of women and girls playing community AF. The study highlights the significant injury burden for women playing AF, with more than half of the cohort reporting a previous significant injury and around one-quarter a current injury. The most prevalent injuries were to the knee, ankle, and hand/finger. Self-reported suspected concussions were also high, with more than one-third of the sample reporting a suspected concussion with LOC. The sizeable injury rates highlight the vital need for more research into injury mechanisms and effective injury prevention strategies for women playing AF.

## Supplemental Material

sj-docx-1-sph-10.1177_19417381241303512 – Supplemental material for Head, Hands, Knees and Ankles, Knees and Ankles: Injury Profiles of Women and Girls Playing Community Australian Football Supplemental material, sj-docx-1-sph-10.1177_19417381241303512 for Head, Hands, Knees and Ankles, Knees and Ankles: Injury Profiles of Women and Girls Playing Community Australian Football by Sallie M. Cowan, Brooke E. Patterson, Matthew G. King, Mick A. Girdwood, Andrea B. Mosler, Alex Donaldson, Adam G. Culvenor, Andrea M. Bruder, Melissa J. Haberfield, Michael Makdissi, Christian J. Barton, Eliza Roughead, Sarah Lampard, Karina Chilman, Christian Bonello, Elizabeth Birch, Joshua Frost, Deirdre E. McGhee and Kay M. Crossley in Sports Health

sj-pdf-2-sph-10.1177_19417381241303512 – Supplemental material for Head, Hands, Knees and Ankles, Knees and Ankles: Injury Profiles of Women and Girls Playing Community Australian Football Supplemental material, sj-pdf-2-sph-10.1177_19417381241303512 for Head, Hands, Knees and Ankles, Knees and Ankles: Injury Profiles of Women and Girls Playing Community Australian Football by Sallie M. Cowan, Brooke E. Patterson, Matthew G. King, Mick A. Girdwood, Andrea B. Mosler, Alex Donaldson, Adam G. Culvenor, Andrea M. Bruder, Melissa J. Haberfield, Michael Makdissi, Christian J. Barton, Eliza Roughead, Sarah Lampard, Karina Chilman, Christian Bonello, Elizabeth Birch, Joshua Frost, Deirdre E. McGhee and Kay M. Crossley in Sports Health

sj-pdf-3-sph-10.1177_19417381241303512 – Supplemental material for Head, Hands, Knees and Ankles, Knees and Ankles: Injury Profiles of Women and Girls Playing Community Australian Football Supplemental material, sj-pdf-3-sph-10.1177_19417381241303512 for Head, Hands, Knees and Ankles, Knees and Ankles: Injury Profiles of Women and Girls Playing Community Australian Football by Sallie M. Cowan, Brooke E. Patterson, Matthew G. King, Mick A. Girdwood, Andrea B. Mosler, Alex Donaldson, Adam G. Culvenor, Andrea M. Bruder, Melissa J. Haberfield, Michael Makdissi, Christian J. Barton, Eliza Roughead, Sarah Lampard, Karina Chilman, Christian Bonello, Elizabeth Birch, Joshua Frost, Deirdre E. McGhee and Kay M. Crossley in Sports Health
