# Supplementary material for: Head, Hands, Knees and Ankles, Knees and Ankles: Injury Profiles of Women and Girls Playing Community Australian Football
Source: Sports Health. 2025 Feb 4;17(5):1101–10. doi: 10.1177/19417381241303512 (PMC11795580; doi:10.1177/19417381241303512)
Supplement: sj-docx-1-sph-10.1177_19417381241303512 – Supplemental material for Head, Hands, Knees and Ankles, Knees and Ankles: Injury Profiles of Women and Girls Playing Community Australian Football [file sj-docx-1-sph-10.1177_19417381241303512.docx]

| ***Item Category*** | ***Checklist Item*** | ***Page no.*** | ***Description*** |
| --- | --- | --- | --- |
| **Design** | Study Design | 6 | This was a cross-sectional survey (December 2020 – May 2021) administered before the interventions for a stepped-wedge, cluster randomised trial measuring the effectiveness of an injury prevention program for women and girls playing community AF |
| **Ethics** | Ethics approval | 6 | Ethical approval was granted by La Trobe Human Research Ethics Committee (HEC 20488) |
|  | Informed consent | 6 | Players were invited to complete the survey by clicking on a link distributed by their coaches. Players provided electronic consent by clicking on the ‘I AGREE’ button after reading the Participant Information sheet which informed participants that the initial questionnaire will take 5-10 minutes to complete, how and where and how long the data will be stored, who the lead investigator was, and the purpose of the study. All surveys were collected via the secure Redcap platform |
|  | Data protection | 7 | The REDCap data collection platform is a secure and customisable web-based application (https://www.project-redcap.org/) |
| **Development and testing** | Development and testing | 7 | The baseline player survey collected player demographics, football experience, injury, medical and sporting history (Appendix 2). The survey was developed based on available literature and was piloted on approximately 20 Australian football players prior to implementation. |
| **Recruitment process** | Survey type |  | The data was collected using a closed survey, participants were required to provide informed consent prior to gaining access to the survey. |
|  | Contact mode | 6 | Players were informed about the study by their coach who then forwarded them a weblink. |
|  | Advertising the survey | 6 | Players who did not reply to the initial weblink sent by the coach, were reminded by the coach and team manager. They were provided with the contact details of the research team and invited to get in touch with the research team, a member of the research team also attended training to inform players of the study. Leading officials, players, coaches, and partner organisations supported recruitment via social and mainstream media. |
| **Survey Administration** | Web/E-mail | 7 | Surveys were collected via the secure online platform REDCap (https://www.project-redcap.org/) |
|  | Context | 6 | The survey link was provided to players via their coach, the link took players to the Participant Information Sheet which detailed the project upon clicking ‘I AGREE’ players were directed straight to the online survey. |
|  | Mandatory/voluntary | 6 | Voluntary completion of the survey once the player had consented to the study. |
|  | Incentives |  | No incentives were provided. |
|  | Time/Date | 6 | Responses were collected over the two football seasons (April – September) of the study in 2021 and 2022 |
|  | Randomization of items or questionnaires |  | No randomisation of items was used. |
|  | Adaptive questioning |  | Adaptive questioning (branched) was used. Relevant survey items were displayed based on previous responses. |
|  | Number of Items |  | There were a maximum of 44 items, although because of the adaptive nature of the survey, not all participants answered all items. |
|  | Number of screens (pages) |  | The questionnaire was displayed on one screen. |
|  | Completeness check |  | Almost all survey items were deemed mandatory, with respondents prompted to complete outstanding items before leaving the survey. Most items except the adaptive questioning included a ‘Prefer not to say’ option. |
|  | Review step |  | Players were not able to review their answers after submitting their responses. |
| **Response rate** | Unique site visitor |  | Only valid, non-duplicated entries were collated with a system generated record number. |
|  | View rate (Ratio of unique survey visitors/unique site visitors) |  | View rate was unable to be collected on the platform |
|  | Participation rate (Ratio of unique visitors who agreed to participate/unique first survey page visitors) | 6, 9 | There were 5182 registered players were invited and 2435 completed the survey for this study, giving a participation rate of 47%. |
|  | Completion rate (Ratio of users who finished the survey/users who agreed to participate) | 9 | Of the 2574 players who consented to participate in the trial 2435 completed the survey for this study, giving a completion rate of 95%. |
| **Preventing multiple entries from the same individual** | Cookies used |  | Cookies were not used |
|  | IP check |  | IP addresses were not collated |
|  | Log file analysis |  | The study did not include a log file analysis. Duplicate entries were assessed via participants name and email address, where duplicates were completed, the complete entry was used for analysis |
|  | Registration |  | This was a closed survey; entry was provided via informed consent |
| **Analysis** | Handling of incomplete questionnaires |  | Only completed questionnaires were included in the final dataset. |
|  | Questionnaires submitted with an atypical timestamp |  | N/A |
|  | Statistical correction |  | N/A |
