# Supplementary material for: Head, Hands, Knees and Ankles, Knees and Ankles: Injury Profiles of Women and Girls Playing Community Australian Football
Source: Sports Health. 2025 Feb 4;17(5):1101–10. doi: 10.1177/19417381241303512 (PMC11795580; doi:10.1177/19417381241303512)
Supplement: sj-pdf-2-sph-10.1177_19417381241303512 – Supplemental material for Head, Hands, Knees and Ankles, Knees and Ankles: Injury Profiles of Women and Girls Playing Community Australian Football [file sj-pdf-2-sph-10.1177_19417381241303512.pdf]

PLAYER BASELINE QUESTIONS

PLAYER BASELINE QUESTIONNAIRE

What is the postcode of your home address?

____

What is your year of birth?

____

How would you describe your gender identity? (Select all that apply)

◯ Woman ◯ Man ◯ Non-binary ◯ Other (please specify below *) ◯ I do not wish to disclose

Please specify

____

Which is your preferred kicking leg?

◯ Right
◯ Left

What is your height (cm)? (estimate if you don't know exactly)

Leave blank if prefer not to say

____

What is your weight (kg)? (estimate if you don't know exactly)

Leave blank if prefer not to say

____

Are you of Aboriginal and /or Torres Strait Islander origin?

◯ Yes
◯ No

What is your normal occupation?
(put student if primarily studying or unemployed or on leave if not working)

____

What is your current employment status? (select any that apply)

☐ Working full time
☐ Working part time
☐ Not working
☐ Home duties
☐ Full time student
☐ Part time student
☐ Other

What is your highest level of education you have completed?

◯ Current school student (including VCAL)
◯ Less than Year 12 or equivalent
◯ Year 12 or equivalent (VCE, HSC, Leaving certificate)
◯ Vocational/technical/trade school
◯ Bachelor degree
◯ Post-graduate degree

FOOTBALL INFORMATION

What club do you currently play with? (e.g. Darebin)

If you play for more than one team, please choose the team you predominantly play with. Answer all the questions below in respect to this team.

____

Team/ Age Group

- ◯ Under 16s
- ◯ Under 17s/18s/19s/Youth
- ◯ Womens

League

- ◯ Eastern Football Netball League
- ◯ Victorian Amateur Football Association
- ◯ Essendon District Football League
- ◯ Yarra Junior Football League
- ◯ Barwon Football League (Juniors/Seniors)
- ◯ Goldfields (includes Ballarat Football Netball, Riddell District Football Netball)
- ◯ Western Region Football League
- ◯ South East (Juniors/Seniors)
- ◯ Southern Metro Junior Football League
- ◯ Southern Gippsland (Juniors/Seniors)
- ◯ Southern Football Netball League
- ◯ Bendigo (Bendigo Juniors, AFL Central Victoria Womens League)
- ◯ Northern Football Netball League
- ◯ Other

Please specify the league

____

Competition

- ◯ Division 1
- ◯ Division 2
- ◯ Division 3
- ◯ Division 4
- ◯ Premier A
- ◯ Premier B
- ◯ Premier C
- ◯ Other (please specify)

Please specify competition

____

Do you currently play for a second club/school?

- ◯ Yes
- ◯ No

Approximately how many games in total have you played in your career thus far?

- ◯ 0-10
- ◯ 11-50
- ◯ 51-100
- ◯ 101-150
- ◯ 151+

What is the highest level you have played?

- ◯ National
- ◯ State
- ◯ Community/Local/School

What position do you usually play?

- ◯ Midfielder
- ◯ Defender
- ◯ Forward
- ◯ Ruck

Have you played any other competitive sport?

- ◯ Yes
- ◯ No

Which sports and at what level

	International	National	State	Local/community/ school
Other football code (e.g. soccer, rugby)	☐	☐	☐	☐
Netball, Basketball, Volleyball, Hockey	☐	☐	☐	☐
Cricket, Tennis	☐	☐	☐	☐
Water sports, Athletics, Cycling	☐	☐	☐	☐
Other	☐	☐	☐	☐

INURY/ HEALTH HISTORY

Have you ever had an injury that resulted in you missing football (or other sport) training/games for more than a month (4 weeks)?

◯ Yes ◯ No

What was the location of the injury(ies)?

- ☐ Head
- ☐ Neck
- ☐ Shoulder
- ☐ Elbow
- ☐ Hand/fingers
- ☐ Back
- ☐ Hip/groin
- ☐ Quadriceps
- ☐ Hamstring
- ☐ Knee
- ☐ Ankle
- ☐ Calf
- ☐ Leg/shin
- ☐ Foot

What year* did you sustain your most recent knee injury? *Include 2021

____

What year* did you sustain your most recent head injury? *Include 2021

____

Have you ever had an anterior cruciate ligament (ACL) injury?

- ☐ No
- ☐ Yes, right knee
- ☐ Yes, left knee

Did you have ACL reconstruction surgery? (tick all that apply)

☐ No
☐ Yes left knee
☐ Yes right knee

How many reconstructions have you had? ○ 1 ○ 2 ○ 3 ○ 4 ○ 5 ○ 6

What year* did you sustain your most recent ACL injury? *Include 2021 ____________________

Has anyone else in your immediate family had an ACL injury? (biological mother, father, brother, sister) ○ No ○ Yes ○ Unsure

SERIOUS CONCUSSION HISTORY

	0	1	2	3	4	5	6	7	8	9	10
How many times have you been knocked out cold or unconscious during football?	○	○	○	○	○	○	○	○	○	○	○
How many times have you been knocked out cold or unconscious during any other sport apart from football	○	○	○	○	○	○	○	○	○	○	○
How many times have you been knocked out cold or unconscious that was non-sport related?	○	○	○	○	○	○	○	○	○	○	○

MILDER CONCUSSION

^where you were briefly dazed or stunned after a collision or where you had other symptoms (such as dizziness, confusion, balance problems, blurred vision, slowed reactions, nausea, difficulty concentrating or headache) after a collision or impact

	0	1	2	3	4	5	6	7	8	9	10
How many football-related milder concussions^ have you had?	○	○	○	○	○	○	○	○	○	○	○
How many milder concussions^ have you had during other sports other than football?	○	○	○	○	○	○	○	○	○	○	○
How many non-sport-related milder concussions^ have you had?	○	○	○	○	○	○	○	○	○	○	○

What year* did you sustain your most recent (football related) concussion? *Include 2021 ____________________

Do you currently (during the last week) have any injury (including concussion), and/or pain that has limited your full participation in training or games?

○ Yes
○ No

Please list all injuries/ pains and which body part(s) are currently affected	☐ Head<br>☐ Neck<br>☐ Shoulder<br>☐ Elbow<br>☐ Hand/fingers<br>☐ Back<br>☐ Hip/groin<br>☐ Quadriceps<br>☐ Hamstring<br>☐ Knee<br>☐ Ankle<br>☐ Calf<br>☐ foot
Do you have any of the following conditions (tick all that apply)	☐ diabetes<br>☐ asthma<br>☐ heart condition<br>☐ high blood pressure<br>☐ lung problems (other than asthma)<br>☐ polycystic ovary syndrome<br>☐ endometriosis<br>☐ cancer<br>☐ none<br>☐ prefer not to say
Do you currently follow a specific injury prevention program?	◯ Yes<br>◯ No
Please describe the injury prevention program you follow	◯ Prep-To-Play<br>◯ Footy First<br>◯ FIFA 11+<br>◯ Other *
*Please describe the injury prevention program you follow	________________________
Do you have a regular menstrual cycle (10-12 cycles per year)?	◯ Yes ◯ No ◯ Prefer not to say
Do you use contraceptive medication (eg pill, implant, injection)?	◯ Yes ◯ No ◯ Prefer not to say
Are you undertaking any hormone therapy other than contraceptive medication (eg for IVF, gender transition, early menopause, endometriosis)?	◯ Yes ◯ No ◯ Prefer not to say
Are you currently breastfeeding?	◯ No ◯ Yes ◯ Pregnant<br>◯ Prefer not to say
Have you ever had a direct blow to your breast (i.e. from other athlete, the ball or the ground) that resulted in: Tick all that apply;	☐ Breast pain<br>☐ Breast swelling<br>☐ Breast bruising<br>☐ Decreased ability to perform my sport because of this breast injury<br>☐ I have never had a direct blow to my breast<br>☐ Other (describe)
Please describe your breast injury	________________________

Are you currently pregnant? ◯ No ◯ Yes ◯ Prefer not to say
