# Supplementary material for: Head, Hands, Knees and Ankles, Knees and Ankles: Injury Profiles of Women and Girls Playing Community Australian Football
Source: Sports Health. 2025 Feb 4;17(5):1101–10. doi: 10.1177/19417381241303512 (PMC11795580; doi:10.1177/19417381241303512)
Supplement: sj-pdf-3-sph-10.1177_19417381241303512 – Supplemental material for Head, Hands, Knees and Ankles, Knees and Ankles: Injury Profiles of Women and Girls Playing Community Australian Football [file sj-pdf-3-sph-10.1177_19417381241303512.pdf]

Appendix 3

Table A1: Self reported injury prevalence by body region. (n=2434 responses)

	Current injury (n = 619, 25.4%)			Previous signifcant injury history (n = 1238, 50.9%)		
	n	% (of current)	95% CI	n	% (of signifcant)	95% CI
Head	53	8.6	6.5 – 11.0%	138	11.1	9.4 – 13.0%
Neck	39	6.3	4.5 – 8.5%	31	2.5	1.7 – 3.5%
Shoulder	68	11.0	8.6 – 13.7%	142	11.5	9.7 – 13.4%
Elbow	8	1.3	0.6 – 2.5%	40	3.2	2.3 – 4.4%
Hand / Fingers	100	16.1	13.3 – 19.3%	317	25.6	23.2 – 28.1%
Back	81	13.1	10.5 – 16.0%	113	9.1	7.6 – 10.9%
Hip / Groin	52	8.4	6.3 – 10.9%	80	6.5	5.2 – 8.0%
Quad	40	6.5	4.7 – 8.7%	38	3.1	2.2 – 4.2%
Hamstring	42	6.8	4.9 – 9.1%	75	6.1	4.8 – 7.5%
Knee	160	25.8	22.4 – 29.5%	403	32.6	29.9 – 35.2%
Ankle	130	21.0	17.8 – 24.4%	427	34.5	31.8 – 37.2%
Calf	100	16.1	13.3 – 19.3%	63	5.1	3.9 – 6.5%
Foot	54	8.7	6.6 – 11.2%	109	8.7	7.2 – 10.4%

Table A2: Results summary from univariate logistic regression models for ACL injury history.

Term	Estimate	SE	Z-Statistic	p value	Odds ratio	95% CI
Age	0.07	0.01	6.06	0	1.07	1.05 to 1.09
BMI	0.03	0.02	1.7	0.09	1.03	1 to 1.07
Concussion History	0.12	0.18	0.66	0.51	1.12	0.8 to 1.58
SEIFA Add/Dis Decile* - Linear	-0.47	0.36	-1.33	0.19	0.62	0.31 to 1.26
SEIFA Add/Dis Decile* - Quadratic	0.04	0.34	0.11	0.91	1.04	0.53 to 2.03
SEIFA Add/Dis Decile* - Cubic	0.08	0.37	0.23	0.82	1.09	0.53 to 2.25
SEIFA Add/Dis Decile* - ^4	0	0.35	0.01	1	1	0.51 to 1.97
SEIFA Add/Dis Decile* - ^5	-0.07	0.35	-0.21	0.83	0.93	0.47 to 1.83
SEIFA Add/Dis Decile* - ^6	0.18	0.35	0.5	0.61	1.2	0.6 to 2.4
SEIFA Add/Dis Decile* - ^7	-0.1	0.37	-0.27	0.78	0.9	0.44 to 1.86
SEIFA Add/Dis Decile* - ^8	0.87	0.37	2.35	0.02	2.38	1.15 to 4.92
SEIFA Add/Dis Decile* - ^9	-0.27	0.32	-0.83	0.41	0.77	0.41 to 1.44
Location (Regional)	0	0	-1.1	0.27	1	1 to 1
Games played* - Linear	0.1	0.18	0.56	0.57	1.11	0.77 to 1.59
Games played* - Quadratic	0.8	0.3	2.69	0.01	2.22	1.24 to 3.97
Games played* - Cubic	0.61	0.28	2.19	0.03	1.85	1.07 to 3.2
Games played* - ^4	0.13	0.29	0.44	0.66	1.14	0.64 to 2.02
Highest level played* - Linear	-0.28	0.26	-1.07	0.29	0.75	0.45 to 1.27
Highest level played* - Quadratic	-0.51	0.38	-1.33	0.18	0.6	0.29 to 1.27
Played Other Sport	-0.33	0.28	-1.16	0.25	0.72	0.41 to 1.25
Played Other football code	0.15	0.28	0.55	0.58	1.17	0.67 to 2.02

* = variables included in model as ordinal factor. SEIFA Add/Dis = Socio-Economic Indexes for Areas relative advantage and disadvantage index.

Table A3: Results summary from univariate logistic regression models for serious knee injury history.

Term	Estimate	SE	Z-Statistic	p value	Odds ratio	95% CI
Age	0.03	0.01	3.9	0	1.03	1.01 to 1.05
BMI	0.03	0.01	2.85	0	1.03	1.01 to 1.06
Concussion History	0.18	0.11	1.68	0.09	1.2	0.97 to 1.48
SEIFA Add/Dis Decile* - Linear	-0.39	0.23	-1.73	0.08	0.68	0.43 to 1.05
SEIFA Add/Dis Decile* - Quadratic	0.15	0.21	0.72	0.47	1.17	0.77 to 1.77
SEIFA Add/Dis Decile* - Cubic	-0.11	0.24	-0.47	0.64	0.89	0.56 to 1.42
SEIFA Add/Dis Decile* - ^4	0	0.22	0	1	1	0.64 to 1.55
SEIFA Add/Dis Decile* - ^5	-0.13	0.21	-0.61	0.54	0.88	0.59 to 1.32
SEIFA Add/Dis Decile* - ^6	0.19	0.23	0.83	0.41	1.21	0.77 to 1.9
SEIFA Add/Dis Decile* - ^7	-0.36	0.25	-1.46	0.15	0.7	0.43 to 1.13
SEIFA Add/Dis Decile* - ^8	0.44	0.23	1.93	0.05	1.55	0.99 to 2.43
SEIFA Add/Dis Decile* - ^9	-0.3	0.2	-1.49	0.14	0.74	0.5 to 1.1
Location (Regional)	0	0	-1.15	0.25	1	1 to 1
Games played* - Linear	-0.02	0.12	-0.14	0.89	0.98	0.79 to 1.23
Games played* - Quadratic	0.73	0.21	3.4	0	2.07	1.36 to 3.15
Games played* - Cubic	0.29	0.19	1.51	0.13	1.34	0.92 to 1.95
Games played* - ^4	0.21	0.2	1.05	0.29	1.23	0.83 to 1.82
Highest level played* - Linear	0	0.16	0.02	0.98	1	0.73 to 1.38
Highest level played* - Quadratic	-0.64	0.24	-2.64	0.01	0.53	0.33 to 0.85
Played Other Sport	-0.05	0.19	-0.26	0.79	0.95	0.66 to 1.38
Played Other football code	0.31	0.18	1.75	0.08	1.36	0.96 to 1.93

* = variables included in model as ordinal factor. SEIFA Add/Dis = Socio-Economic Indexes for Areas relative advantage and disadvantage index.

Table A4: Results summary from univariate logistic regression models for concussion history.

Term	Estimate	SE	Z-Statistic	p value	Odds ratio	95% CI
Age	0.01	0.01	1.12	0.26	1.01	0.99 to 1.02
BMI	0	0.01	-0.44	0.66	1	0.98 to 1.02
SEIFA Add/Dis Decile* - Linear	0.06	0.19	0.3	0.76	1.06	0.73 to 1.54
SEIFA Add/Dis Decile* - Quadratic	-0.05	0.18	-0.26	0.79	0.95	0.68 to 1.35
SEIFA Add/Dis Decile* - Cubic	-0.1	0.2	-0.51	0.61	0.9	0.61 to 1.34
SEIFA Add/Dis Decile* - ^4	0.23	0.19	1.24	0.21	1.26	0.87 to 1.83
SEIFA Add/Dis Decile* - ^5	-0.2	0.16	-1.25	0.21	0.82	0.6 to 1.12
SEIFA Add/Dis Decile* - ^6	0.02	0.19	0.09	0.93	1.02	0.7 to 1.48
SEIFA Add/Dis Decile* - ^7	0.18	0.21	0.86	0.39	1.2	0.79 to 1.82
SEIFA Add/Dis Decile* - ^8	-0.15	0.19	-0.8	0.42	0.86	0.6 to 1.24
SEIFA Add/Dis Decile* - ^9	-0.1	0.16	-0.64	0.53	0.9	0.66 to 1.23
Location (Regional)	0	0	-0.06	0.95	1	1 to 1
Games played* - Linear	0.23	0.09	2.61	0.01	1.26	1.06 to 1.5
Games played* - Quadratic	0.55	0.19	2.84	0	1.74	1.19 to 2.54
Games played* - Cubic	-0.04	0.17	-0.21	0.83	0.96	0.69 to 1.35
Games played* - ^4	0.18	0.17	1.1	0.27	1.2	0.87 to 1.67
Highest level played* - Linear	0.22	0.13	1.68	0.09	1.25	0.96 to 1.61
Highest level played* - Quadratic	-0.5	0.23	-2.18	0.03	0.61	0.39 to 0.95
Played Other Sport	-0.26	0.17	-1.53	0.12	0.77	0.55 to 1.08
Played Other football code	0.46	0.13	3.53	0	1.59	1.23 to 2.05

* = variables included in model as ordinal factor. SEIFA Add/Dis = Socio-Economic Indexes for Areas relative advantage and disadvantage index.
